# Death of Dr. Harlan

**Published:** 1843-10-28

**Authors:** 


### DEATH OF DR. HARLAN.

We regret to announce the death of Dr. Richard Harlan, late of this city, who died recently of apoplexy at New Orleans, whither he had lately removed. As a man of genuine science, few in the profession of our country could compete with Dr. H. As a naturalist he ranked deservedly high both at home and abroad. A resident of Paris at the time his valuable collection of Comparative Anatomy was destroyed in this country, duplicates of many of the finest specimens at the Jardin des Plantes were immediately presentedto him. Dr. Harlan was one of the earliest contributors to this journal; a number of valuable communications were furnished by him during his residence abroad.

